# The TcEG1 beetle (*Tribolium castaneum*) cellulase produced in transgenic switchgrass is active at alkaline pH and auto-hydrolyzes biomass for increased cellobiose release

**DOI:** 10.1186/s13068-017-0918-6

**Published:** 2017-11-30

**Authors:** Jonathan D. Willis, Joshua N. Grant, Mitra Mazarei, Lindsey M. Kline, Caroline S. Rempe, A. Grace Collins, Geoffrey B. Turner, Stephen R. Decker, Robert W. Sykes, Mark F. Davis, Nicole Labbe, Juan L. Jurat-Fuentes, C. Neal Stewart

**Affiliations:** 1 Department of Plant Sciences, University of Tennessee, Knoxville, TN 37996 USA; 2 Center for Renewable Carbon, University of Tennessee, Knoxville, TN 37996 USA; 3 The National Research Energy Laboratory, Golden, CO 80401 USA; 4 Department of Entomology and Plant Pathology, University of Tennessee, Knoxville, TN 37996 USA; 5 UT-ORNL Graduate School of Genome Science and Technology, University of Tennessee, Knoxville, TN 37996 USA; 6 BioEnergy Science Center, Oak Ridge National Laboratory, Oak Ridge, TN 37831 USA

**Keywords:** Switchgrass, Auto-hydrolysis, Glycosyl hydrolase, β-1,4-Endoglucanase, Insect, Cellulase, Biofuel, *Tribolium castaneum*

## Abstract

**Background:**

Genetically engineered biofuel crops, such as switchgrass (*Panicum virgatum* L.), that produce their own cell wall-digesting cellulase enzymes would reduce costs of cellulosic biofuel production. To date, non-bioenergy plant models have been used in nearly all studies assessing the synthesis and activity of plant-produced fungal and bacterial cellulases. One potential source for cellulolytic enzyme genes is herbivorous insects adapted to digest plant cell walls. Here we examine the potential of transgenic switchgrass-produced TcEG1 cellulase from *Tribolium castaneum* (red flour beetle). This enzyme, when overproduced in *Escherichia coli* and *Saccharomyces cerevisiae*, efficiently digests cellulose at optima of 50 °C and pH 12.0.

**Results:**

TcEG1 that was produced in green transgenic switchgrass tissue had a range of endoglucanase activity of 0.16–0.05 units (µM glucose release/min/mg) at 50 °C and pH 12.0. TcEG1 activity from air-dried leaves was unchanged from that from green tissue, but when tissue was dried in a desiccant oven (46 °C), specific enzyme activity decreased by 60%. When transgenic biomass was “dropped-in” into an alkaline buffer (pH 12.0) and allowed to incubate at 50 °C, cellobiose release was increased up to 77% over non-transgenic biomass. Saccharification was increased in one transgenic event by 28%, which had a concurrent decrease in lignin content of 9%. Histological analysis revealed an increase in cell wall thickness with no change to cell area or perimeter. Transgenic plants produced more, albeit narrower, tillers with equivalent dry biomass as the control.

**Conclusions:**

This work describes the first study in which an insect cellulase has been produced in transgenic plants; in this case, the dedicated bioenergy crop switchgrass. Switchgrass overexpressing the TcEG1 gene appeared to be morphologically similar to its non-transgenic control and produced equivalent dry biomass. Therefore, we propose TcEG1 transgenics could be bred with other transgenic germplasm (e.g., low-lignin lines) to yield new switchgrass with synergistically reduced recalcitrance to biofuel production. In addition, transgenes for other cell wall degrading enzymes may be stacked with TcEG1 in switchgrass to yield complementary cell wall digestion features and complete auto-hydrolysis.

**Electronic supplementary material:**

The online version of this article (doi:10.1186/s13068-017-0918-6) contains supplementary material, which is available to authorized users.

## Background

One overriding goal in bioenergy is the efficient conversion of biomass into biofuel to replace petroleum. Biomass sources include crop residues such as maize stover and dedicated perennial crops such as switchgrass (*Panicum virgatum*). Dedicated biomass crops are attractive inasmuch as growing demands for fuel might be met by low-input bioenergy crops grown on marginal lands unsuitable for food crop production [1]. Plants utilizing C4 photosynthesis, such as switchgrass, have increased water-use efficiency over C3 plants. Furthermore, switchgrass and other perennial grasses have lower nutrient fertilizer requirements compared with most C4 cereal crops (e.g., maize) [2–4]. For switchgrass-based bioenergy, aboveground biomass would be harvested using standard forage baling equipment at the end of the growing season after the first frost in temperate and sub-tropical regions where the feedstock is adapted. This timing allows the plant to remobilize nitrogen and other nutrients to belowground biomass, thereby endowing high nutrient use efficiency. Utilization of farmer contracts from biorefineries would allow farmers to ‘permanently’ install switchgrass at low risk [5, 6]. Production of perennial, dedicated cellulosic feedstocks on marginal lands will allow farmers to produce a profitable and environmentally stable fuel source [1].

Enzyme cost is significant for current cellulosic ethanol production. High titers of expensive cellulase cocktails are required to convert recalcitrant plant cell walls into simple sugars for fermentation. Economic modeling has demonstrated that cellulosic ethanol refineries should use an integrated approach of on-site feedstock and cellulase production to reduce total cost [7]. A biofuel feedstock that simultaneously produces its own cocktail of cellulolytic enzymes has been proposed as an all-in-one model integrated system for reducing enzyme costs [8, 9]. There are several challenges to address in designing such a feedstock. First, complete digestion of cellulose in the plant cell wall requires the synergistic actions of three types of glycosyl hydrolases (commonly referred to as cellulase enzymes): endoglucanases, exoglucanases, and β-glucosidases [10, 11]. Internal cellulose bonds are broken by endoglucanases [10–12]. Unbound chain ends of cellulose are cleaved by exoglucanases (also called cellobiohydrolases), which release the base units of cellulose, cellobiose. Cellobiose consists of two inverted glucose units, which are broken into free glucose by β-glucosidases. Second, genetically engineered feedstocks would conceivably require the concerted synthesis of each type of enzyme for complete digestion, while not affecting plant growth. Third is the translation of studies in easy-to-transform model plants to bioenergy feedstocks, which has rarely been pursued. Fourth, and very important is choosing the best-suited genes to express in plants for auto-hydrolysis. To date, all cellulase genes engineered into plants are from either bacterial or fungal origins [13].

One intriguing bioprospecting source for biocatalytic enzymes is herbivorous insects [13]. Until recently, it was believed that insect genomes harbor few cellulolytic enzyme-coding genes, but that plant cell walls were largely digested by insect gut symbionts. While symbionts do play a role in the digestion of biomass, increasing evidence from insect genomic and proteomic analyses reveals that insects indeed produce endogenous cellulolytic enzymes [14–16]. Insect cellulases should be explored for heterologous production in plant hosts, for various reasons, which include cases in which cellulases require temperature optima from 40 to 65 °C and alkaline pH optima [17–19].

Here, we report on transgenic switchgrass that overexpresses a gene encoding TcEG1, an endoglucanase produced in the digestive system of the red flour beetle (*Tribolium castaneum*). Our goal was to assess the potential of the transgenic production of the beetle cellulase in switchgrass for biomass degradation under relevant biofuel production conditions.

## Methods

### Vector construction

**Fig. 1:**
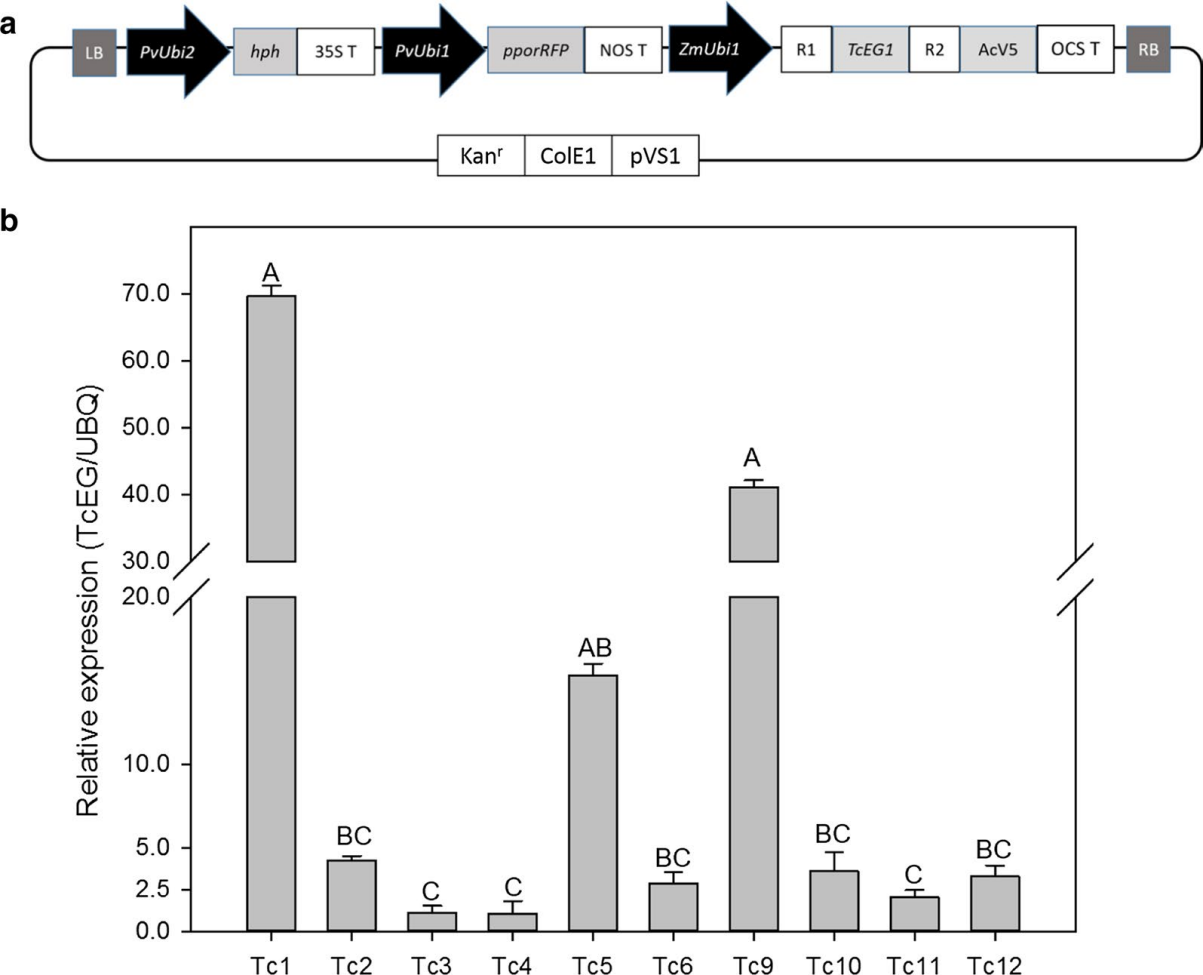
Transformation vector map and relative transcript abundance of TcEG1 in transgenic switchgrass. **a** pANIC-10A-TcEG1 vector used for expression of *TcEG1* in transgenic switchgrass. LB: left border; PvUbi2: switchgrass ubiquitin 2 promoter and intron; hph: hygromycin B phosphotransferase coding region; 35S T: 35S terminator sequence; PvUbi1: switchgrass ubiquitin 1 promoter and intron; pporRFP: *Porites porites* orange fluorescent protein coding region; NOS T: *Agrobacterium tumefaciens nos* terminator sequence; ZmUbi1: maize ubiquitin 1 promoter; R1 and R2: *attR1* and *attR2* recombinase sites 1 and 2; *TcEG1*: *TcEG1* cDNA open reading frame; AcV5: epitope tag; RB: right border; Kan^r^: kanamycin resistance gene; ColE1: origin of replication in *E. coli*; pVS1: origin of replication in *A. tumefaciens*; OCS T: octopine synthase terminator sequence. **b** Relative transcript abundance of *TcEG1* in stem internodes from transgenic events (Tc-1 to Tc-12). Relative expression analysis was determined by qRT-PCR and normalized to switchgrass ubiquitin 1 (*PvUbi1*). Bars represent mean values of three replicates ± standard error. Bars represented by different letters are significantly different as calculated by LSD (*p* ≤ 0.05)

The *TcEG1* open reading frame sequence [19] was amplified by PCR and cloned into the pCR8 entry vector and then Gateway^®^ sub-cloned into the pANIC-10A plant expression vector [20] to yield the pANIC-10A-TcEG1 vector. The expression cassette containing *TcEG1* was 5′ flanked by the constitutive maize ubiquitin 1 promoter (ZmUbi1), and 3′ flanked by the AcV5 epitope tag and the octopine synthase terminator (Fig. 1). The pANIC-10A-TcEG1 also contained cassettes that included a hygromycin selectable marker and an orange fluorescent protein (OFP) reporter gene from the hard coral *Porites porites* (*pporRFP*) [21]. An epi-fluorescence microscope (Olympus stereo microscope SZX12, Olympus America, Centre Valley, PA) having a 535/30 nm excitation filter and 600/50 nm emissions filter was used to track OFP fluorescence during transgenic callus development and to identify individual putative transgenic lines in vitro.

### Transgenic plant production

Seed-derived callus of lowland switchgrass cv. ‘Performer’ was used to generate Type II embryogenic callus [22], which was stably transformed using *Agrobacterium tumefaciens* strain EHA105 harboring the pANIC-10A-TcEG1 expression vector. Transformed calli were grown in LP9 growth medium [23] supplemented with 400 mg/L timentin and 40 mg/L hygromycin for approximately 2 months. Subsequently, transgenic callus was transferred to regeneration medium [22] that was supplemented with 250 mg/L cefotaxime [24]. Ten putatively independent transgenic plants were successfully regenerated, rooted and acclimated as previously described by Burris et al. [23]. Parallel experiments were performed to produce non-transgenic controls. The non-transgenic material was treated identically except it was not transformed with *Agrobacterium* and did not undergo hygromycin selection. The transgenic and control lines were regenerated at the same time and grown in growth chambers under 16 h light/8 h dark cycles at 25 °C until moved to a greenhouse. Fertilizer (0.02% solution of Peter’s soluble 20-20-20) was applied twice per month.

### RNA extraction and qRT-PCR analysis for TcEG1 transcript abundance

Quantitative RT-PCR was performed to estimate *TcEG1* transcript abundance in transgenic T0 and non-transgenic plants. Total RNA was isolated from stem internodes of triplicate tillers at the R1 (reproductive) developmental stage [25] per event using TRI Reagent according to the manufacturer’s instructions (Sigma-Aldrich, St. Louis, MO). Purified RNA was treated with DNase-1 (Promega, Madison, WI) and 3 µg of treated RNA was used to generate cDNA using oligo-dT and Superscript III according to manufacturer’s instructions (Life Technologies, Carlsbad, CA). qRT-PCR analysis was performed with Power SYBR Green PCR master mix (Life Technologies) according to manufacturer’s protocols for optimization of annealing temperature, primer concentration, and cDNA concentration. The optimized qRT-PCR protocol employed a dilution of cDNA 1:100 with thermal cycling at 95 °C for 3 min, and 40 cycle repeats of (95 °C for 10 s and 50.0 °C for 30 s). The *TcEG1* primers were: *TcEG1*_F 5′-CTGGATTACAATGCGGGATTTC-3′ and *AcV5_R* 5′-AGACCAGCCGCTCGCATCTTTCCAAGA-3′. The relative levels of transcripts were normalized to switchgrass ubiquitin 1 (*PvUbi1*) as a reference gene [26] and primers were PvUbi1_F 5′-CAGCGAGGGCTCAATAATTCCA-3′ and PvUbi1_R 5′-TCTGGCGGACTACAATATCCA-3′ [27]. All experiments were carried out in triplicate technical replicates. The differential Ct method was used to measure transcript abundance after normalization to *PvUbi1* according to Schmittgen and Livak [28]. Statistical analysis was performed with triplicate stem internodes averaged from triplicate measuring using SAS^®^ (Version 9.3 SAS Institute Inc., Cary, NC) programming of mixed model ANOVA and least significant difference (LSD) for all quantifiable data.

The TcEG1 protein sequence was aligned against the switchgrass proteome. Since high homology of an heterologously produced enzyme might be confounded with native switchgrass glycosyl hydrolases, a pBLAST search was performed against the switchgrass proteome database (https://phytozome.jgi.doe.gov/pz/portal.html), which revealed 61 targets with no greater than 46% identity match (Additional file 1: Table S1).

### Protein extraction from plants

Proteins were extracted from plant tissue according to Oraby et al. [29] with modifications. Briefly, 100 mg leaf tissue samples from fresh triplicate R1 development stage tillers were ground under liquid nitrogen to a fine powder. For the dry biomass enzyme analysis, triplicate R1 development stage tillers were collected and either air dried in the greenhouse for 2 weeks or placed in a desiccant oven at 46 °C for 3 days as described by Hardin et al. [25] and immediately processed when removed from the desiccant oven to prevent rehydration. A protein extraction buffer of 50 mM sodium acetate, pH 5.5, 100 mM NaCl, 10% glycerol, 0.5 M disodium EDTA, 1 mM PMSF, and a 1:200 dilution of Sigma plant proteinase inhibitor (Sigma-Aldrich, St. Louis, MO) was added to the fine powder in a 2 mL centrifuge tube and vortexed for 30 s. Samples were centrifuged at 4 °C for 10 min at 10,000×*g* and the supernatant was transferred to fresh tube. A subsequent centrifugation step was performed when excess extracellular debris was present. The protein concentration of each sample was estimated via Bradford assay using the Pierce Coomassie Protein Assay Reagent (Thermo Fisher, Wilmington, DE) following the manufacturer’s instructions with bovine serum albumin (BSA) as the standard. Samples were stored at 4 °C until ready for downstream assays.

### Endoglucanase activity

Endoglucanase activity of protein extracts from plants was determined using a modified dinitrosalicylic acid (DNSA) assay [30] with carboxymethyl cellulose (CMC) sodium salt (Sigma-Aldrich, St. Louis, MO) as the substrate. Protein samples (10 µg) were added in triplicate to substrate solutions (2% w/v in 50 mM sodium phosphate buffer, pH 12.0) and incubated for 1 h at 50 °C. A modified DNSA reagent containing Rochelle salt [30] was added to the samples to halt enzymatic activity, after which a color change developed at 100 °C for 15 min. Samples were centrifuged at 2000×*g* for 2 min to precipitate any remaining substrate. Supernatants were transferred to polystyrene microplates and spectral absorbance at 595 nm was read on a Synergy HT microplate reader (BioTek, Winooski, VT) using the KC4 software (v. 3.1). Background amounts of native sugars and any possible native cellulases from switchgrass leaves were corrected for by subtracting non-transgenic activity values from transgenic TcEG1 activity values. One unit of cellulolytic activity was defined as the amount of enzyme that produced 1 µmol of reducing sugar (glucose equivalents) per minute at 50 °C at pH 12.0. Specific activities were reported as units per mg of protein and represented averages of three independent replicates. Statistical analysis was performed with triplicate measures of proteins extracted from triplicate fresh leaves using SAS^®^ (Version 9.3 SAS Institute Inc.) programming of mixed model ANOVA and LSD for all quantifiable data. The standard error of the mean was calculated and reported in data displays. *p* values of ≤ 0.05 were considered to be statistically significant.

### Cell wall sugar release

Switchgrass tillers were collected at the R1 developmental stage from triplicate greenhouse-grown plants and air dried for 3 weeks at room temperature before grinding to 1 mm (20 mesh) particle size. Sugar release efficiency was determined via NREL high-throughput sugar release assays on pretreated extractives- and starch-free samples [31, 32]. Glucose and xylose release was determined by colorimetric assays with total sugar release being the sum of glucose and xylose released. Statistical analysis was performed with triplicate measures of biomass collected from triplicate pots using SAS^®^ (Version 9.3 SAS Institute Inc.) programming of mixed model ANOVA and LSD for all quantifiable data.

### Auto-hydrolysis of switchgrass biomass

Biomass from three plants per transgenic event and control plants (R1 whole tillers, ground to size 20 mesh, 1 g) was placed into a 125 mL flask containing 50 mM sodium phosphate buffer pH 12.0, to a 5% solution and incubated at 50 °C with shaking. Aliquots (1 mL) were taken at the initiation of the experiment and 1, 2, 3, 6, 24, 48, and 72-h time points. Each aliquot was centrifuged at 10,000×*g* for 10 min and supernatant was removed and stored at − 20 °C until analyzed for free sugars (cellobiose and glucose) via HPLC [33]. At initiation and 1, 2, 3, and 6-h time points, data were taken in triplicate. After the 6-h time point, sugar release remained unchanged and the later time points were not measured in replicate. Statistical analysis on auto-hydrolysis results was performed using one-way ANOVA with Holm–Sidak method for pairwise comparisons.

### Cell wall lignin content and composition

Switchgrass tillers were collected at the R1 developmental stage from triplicate greenhouse-grown plants and air dried for 3 weeks at room temperature before grinding to 1 mm (20 mesh) particle size. The lignin content and composition were determined by pyrolysis molecular beam mass spectrometry (py-MBMS) on extractives- and starch-free samples via NREL high-throughput assays [34]. Statistical analysis was carried out with triplicate measures of biomass collected from triplicate pots using SAS^®^ (Version 9.3 SAS Institute Inc.) programming of mixed model ANOVA and LSD.

### Cell wall histology and measurements

Stem segment samples were collected from second-to-basal internode from three tillers at the R1 developmental stage and each was immediately placed in 2 mL Eppendorf tubes containing and covered with FAA solution, which is composed of 50% ethanol (95% EtOH), 5% acetic acid, and 4% formaldehyde, in water. Internodes were incubated for 4 days in FAA on a shaker, after which the FAA was discarded and replaced with a 10% EtOH solution. After 2 h of gentle shaking, the 10% EtOH was discarded and replaced with 20% EtOH. At 2-h intervals each, 30%, and 50% EtOH were used as serial replacements, followed by 75% EtOH for a 4-h incubation, which was subsequently replaced by 95% EtOH. A 2-day 95% EtOH incubation was performed with a change of solution midway through the incubation. Infiltration of glycol methacrylate was performed using a JB-4 Embedding Kit (Sigma-Aldrich) following manufacturer’s instructions. Infiltrated samples were placed in molds (Sigma-Aldrich) and embedded under a nitrogen vacuum until hardened. After hardening, stem samples were mounted and sectioned to 5 µm with a glass blade microtome (Sorvall Dupont JB-4 microtome, Newtown, CT). Dark field staining was performed with Pontamine Fast Scarlet 4B, which binds specifically to cellulose [35]. Dark field staining of total cell wall components was performed with Calcofluor White [36]. After staining, bright field and dark field images at multiple objectives were taken on a Zeiss Axioplan 2 compound microscope (Carl Zeiss, Oberkochen, Germany). Slides stained with Pontamine Fast Scarlet 4B were observed under a 543 nm laser and images were obtained using a Leica confocal microscope. For an undetermined reason, event Tc-10 could not be stained at sufficient quality for imaging and was removed from further histological analysis. Images were analyzed using ImageJ [37] software to measure cell area, perimeter, and cell wall thickness both by hand and with a custom-built program using Python and Python Imaging Library. Hand measuring occurred for 100 cell wall segments on three slide sections. Program measuring was conducted for all cell walls on 20 slide sections. The custom program, Python Cell Wall Thickness (pyCWT), was developed for the batch determination of plant cell wall thickness from images (cross-sections of plant stem internodes with fluorescently labeled cell walls). This automated approach of approximating plant cell wall thickness was written in Python (Python Software Foundation, Python Language Reference, version 2.7, https://www.python.org) using functions from the Python Imaging Library (PIL, Secret Labs AB) and the Scientific Python (Scipy) libraries ndimage and misc [38] and includes a graphical user interface (GUI) to easily work with batches of files and adjust image processing parameters. Each image analyzed with pyCWT underwent a series of processing steps that converted the image to grayscale, normalized pixel brightness distribution using a histogram, smoothed with a Gaussian blur, and then converted to black and white pixels based on the mean pixel brightness of the current image. A stepwise example of pyCWT functionality is shown (Additional file 1: Figure S1). A binary opening function with a 3 × 3 matrix over 2 iterations was then used to better differentiate dark and light objects. The image was segmented and objects labeled using the PIL function “measurements.label()”. Labeled pixels were mapped back to their coordinate values and binary erosion was used to get a border within each labeled object, which corresponds to the border of a plant cell. Centroids of labeled objects were found with the PIL function “measurementscenter_of_mass()”. The border coordinate values were used to calculate area, using an implementation of Green’s Theorum by Jamie Bull (Bull posted function 2012), and perimeter, by summing distances between adjacent border coordinates, of each object. A size cutoff of 200% of the average cell area and perimeter was implemented to restrict the program from counting large gaps as cells. The mode for cell wall thickness was the recorded value for each image.

Cell wall thickness was calculated by dilating each labeled object (presumably a plant cell) 1 pixel width at a time while keeping track of the total number of objects. When two objects merge, meaning the total object count decreases by one, the current pixel count is considered the thickness of that cell wall. A distribution of all cell wall thickness in pixels is plotted based on the number of dilations required for objects to merge. The mode of cell wall thickness was recorded and when these values were compared with average thickness from manual measurements with ImageJ, there was no significant difference when compared with a *t* test at *p* < 0.05 (Additional file 1: Figure S2). Statistical analysis was performed on the pyCWT image rendered data using SAS^®^ (Version 9.3 SAS Institute Inc.) programming of mixed model ANOVA with LSD.

### Cellulose crystallinity index

Collected tillers at the R1 developmental stage were ground to ½ mm (40 mesh) particle size and the crystallinity index was measured by Fourier transform infrared (FTIR). Spectra were collected using a diamond crystal of an attenuated total reflectance (ATR) accessory of a Perkin Elmer Spectrum One spectrometer (Waltham, MA). Spectra were collected over the range of 4000–650 cm^−1^ in the absorbance mode, with 1 cm^−1^ resolution and eight scans per spectra. Ten spectra were collected for each sample. The data were then ATR corrected and normalized in the Spectrum One software. The index of crystallinity was calculated by the intensity ratio between the bands at 1422 and 899 cm^−1^, assigned to CH_2_ bending mode and deformation of anomeric CH, respectively [39]. Statistical analysis was achieved with triplicate measures of biomass collected using SAS^®^ (Version 9.3 SAS Institute Inc.) programming of mixed model ANOVA with LSD.

### Plant growth analysis

Transgenic T_0_ and non-transgenic control line plants were divided into triplicate, single-tiller replicates and placed in a random design in the greenhouse. Plants were grown to the R1 developmental stage, then tiller number was tallied per plant. The five tallest tillers for each replicate were used as a representation of aboveground plant height and stem diameter, which was measured with a caliper at 10 cm above potting level at internodes. At the R1 stage, the aboveground biomass was harvested for each plant and air dried in the greenhouse for approximately 2 weeks and biomass tallied. Statistical analysis was performed using SAS^®^ (Version 9.3 SAS Institute Inc.) programming of mixed model ANOVA and LSD.

## Results

### Production of TcEG1 transgenic plants, transgene expression, and cellulase enzyme activity

**Fig. 2:**
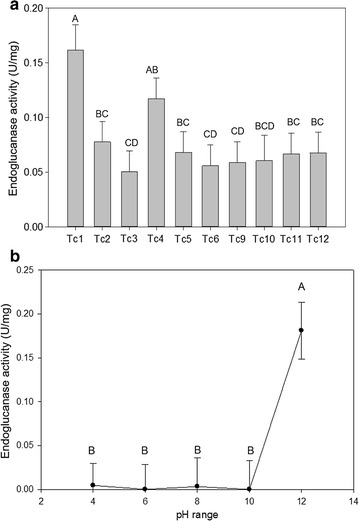
Endoglucanase activity (units/mg of protein) from fresh leaves of transgenic TcEG1 plants. **a** Endoglucanase activity measurement using carboxymethyl cellulose (CMC) as substrate at pH 12.0 on TcEG1 extracted from fresh leaves. Bars represent mean values of three replicates ± standard error for each transgenic event. Bars represented by different letters are significantly different as calculated by LSD (*p* ≤ 0.05). **b** Gradient pH measurement of endoglucanase activity of TcEG1 extracted from fresh leaves of transgenic event Tc-1. Data points represent mean values of three replicates ± standard error. Data points represented by different letters are significantly different as calculated by LSD (*p* ≤ 0.05)

Ten independent transgenic shoots were recovered from ten separate hygromycin-resistant and orange fluorescent callus pieces. Transcript abundance in tillers ranged between 70-fold (relative to *PvUbi1* gene) in event Tc-1 to twofold in Tc-3 (Fig. 1b). All transgenic plants had functionally active TcEG1 endoglucanase as assayed on CMC substrate resulting in increased reduced sugars at 50 °C at pH 12.0 (Fig. 2a). Event Tc-1 had the highest enzyme activity (0.16 ± 0.02 U/mg), whereas event Tc-3 had the lowest activity (0.05 ± 0.02 U/mg; Fig. 2a). In addition, the TcEG1 enzyme activity of event Tc-1 was assessed over a range of pH conditions demonstrating enzymatic activity only at pH 12 (Fig. 2b).

**Fig. 3:**
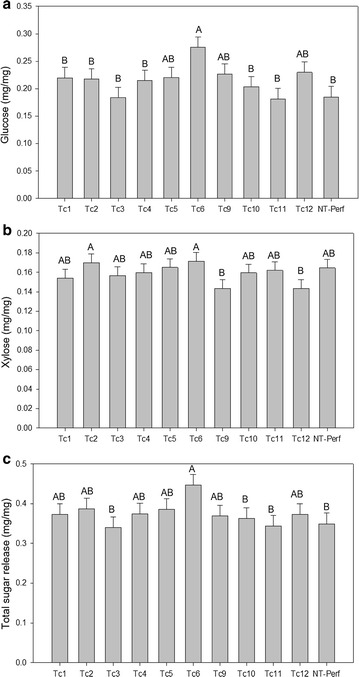
Glucose (**a**), xylose (**b**), and total sugar (**c**) release from transgenic TcEG1 and non-transgenic (NT-Perf) tillers as determined by enzymatic hydrolysis. Bars represent mean values of three replicates ± standard error. Bars represented by different letters are significantly different as calculated by LSD (*p* ≤ 0.05)

High-throughput screening of pretreated, starch-free biomass was used to evaluate the release of soluble sugars. Only event Tc-6 had a significantly higher glucose release (49% higher) than the non-transgenic control (Fig. 3a). There was no difference in xylose release between transgenic and the non-transgenic control (Fig. 3b). Event Tc-6 had significantly higher (28% more) total sugar release relative to the non-transgenic control (Fig. 3c).

**Fig. 4:**
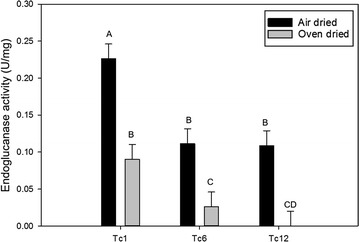
Endoglucanase activity (units/mg of protein) from leaves of three transgenic TcEG1 events using carboxymethyl cellulose (CMC) as substrate at pH 12.0. Leaves were either air dried for 2 weeks in the greenhouse (black bars) or dried for 3 days in an oven at 46 °C (gray bars). Bars represent mean values of three replicates ± standard error. Bars represented by different letters are significantly different as calculated by LSD (*p* ≤ 0.05)

Since commercial switchgrass biomass would be harvested and air dried in the field, it was important to assay endoglucanase activity from dry transgenic switchgrass biomass. We used a subset of transgenic events based on endoglucanase activity and saccharification data with fresh green tissue to test the effect of drying method on the enzyme activity without pretreatment. Cellulolytic activity was maintained after air drying with transgenic event Tc-1 still displaying the highest enzymatic activity (0.23 ± 0.02 U/mg) among all air-dried plants tested (Fig. 4). Only transgenic event, Tc-1, had any discernable enzyme activity after oven drying, but this activity was just 60% of that from air-dried biomass (Fig. 4).

### Auto-hydrolysis of switchgrass biomass

**Fig. 5:**
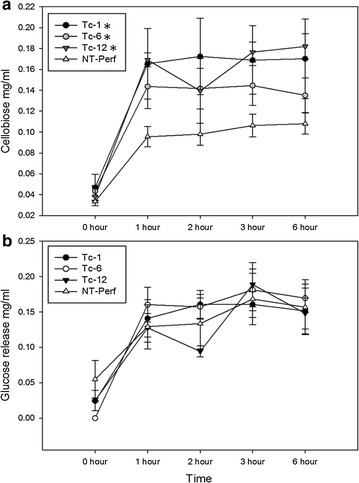
Auto-hydrolysis of TcEG1 switchgrass and non-transgenic switchgrass incubated in alkaline buffer (pH 12.0) at 50 °C. **a** Cellobiose released mg/mL from transgenic *TcEG1* and non-transgenic (NT-Perf) lines over time. **b** Glucose released mg/mL from transgenic *TcEG1* and non-transgenic (NT-Perf) lines over time. Bars represent mean values of three biological replicates ± standard error. Asterisk denotes statistical significant difference of released substrate over time at for event Tc-1 and Tc-12 *p* < 0.001 and Tc-6 *p* = 0.004 using Holm–Sidak method for pairwise comparison for one-way ANOVA with repeated measures

The air-dried switchgrass was analyzed to determine TcEG1 enzyme activity for auto-hydrolysis in an alkaline buffer (pH 12.0 at 50 °C). Transgenic events Tc-1, Tc-6, and Tc-12 all had increased cellobiose release over the course of 6-h compared with non-transgenic biomass (Fig. 5a). The largest change was observed after 1-h of incubation in which cellobiose release from transgenic biomass was increased by 73, 50, and 77% for events Tc-1, Tc-6 and Tc-12, respectively, when compared with the non-transgenic control. Glucose release from transgenic events was equivalent to that of non-transgenic biomass over the course of the experiment (Fig. 5b).

### The effects of TcEG1 production on lignin

**Fig. 6:**
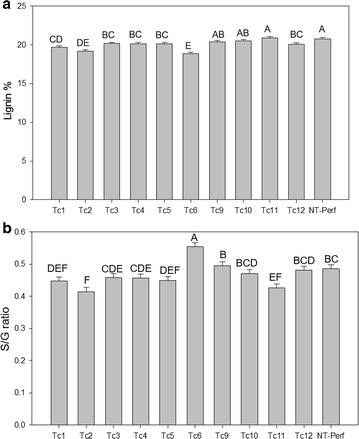
Lignin content (**a**) and S/G ratio (**b**) of transgenic TcEG1 and non-transgenic (NT-Perf) tillers as determined by Py-MBMS. Bars represent mean values of three replicates ± standard error. Bars represented by different letters are significantly different as calculated by LSD (*p* ≤ 0.05)

While there is no a priori reason that TcEG1 synthesis would affect lignification of cell walls, we routinely analyze lignin composition and content for all transgenic feedstock studies given the importance of the polymer in cell wall recalcitrance [40]. Lignin content decreased by up to 9% in events Tc-1, Tc-2, Tc-3, Tc -4, Tc -5, Tc -6, Tc -12, whereas in events Tc-9, Tc-10, and Tc -11 lignin content was equivalent to the control (Fig. 6a). Event Tc-6 had an increased S/G ratio by up to 14%, whereas events Tc-1, Tc-2, Tc-5, and Tc-11 had a decreased S/G ratio by up to 7% relative to the control. The S/G ratio was unchanged in events Tc-3, Tc-4, Tc-9, Tc-10, and Tc-12 compared with the control (Fig. 6b).

### Cell wall architecture and cellulose crystallinity

**Fig. 7:**
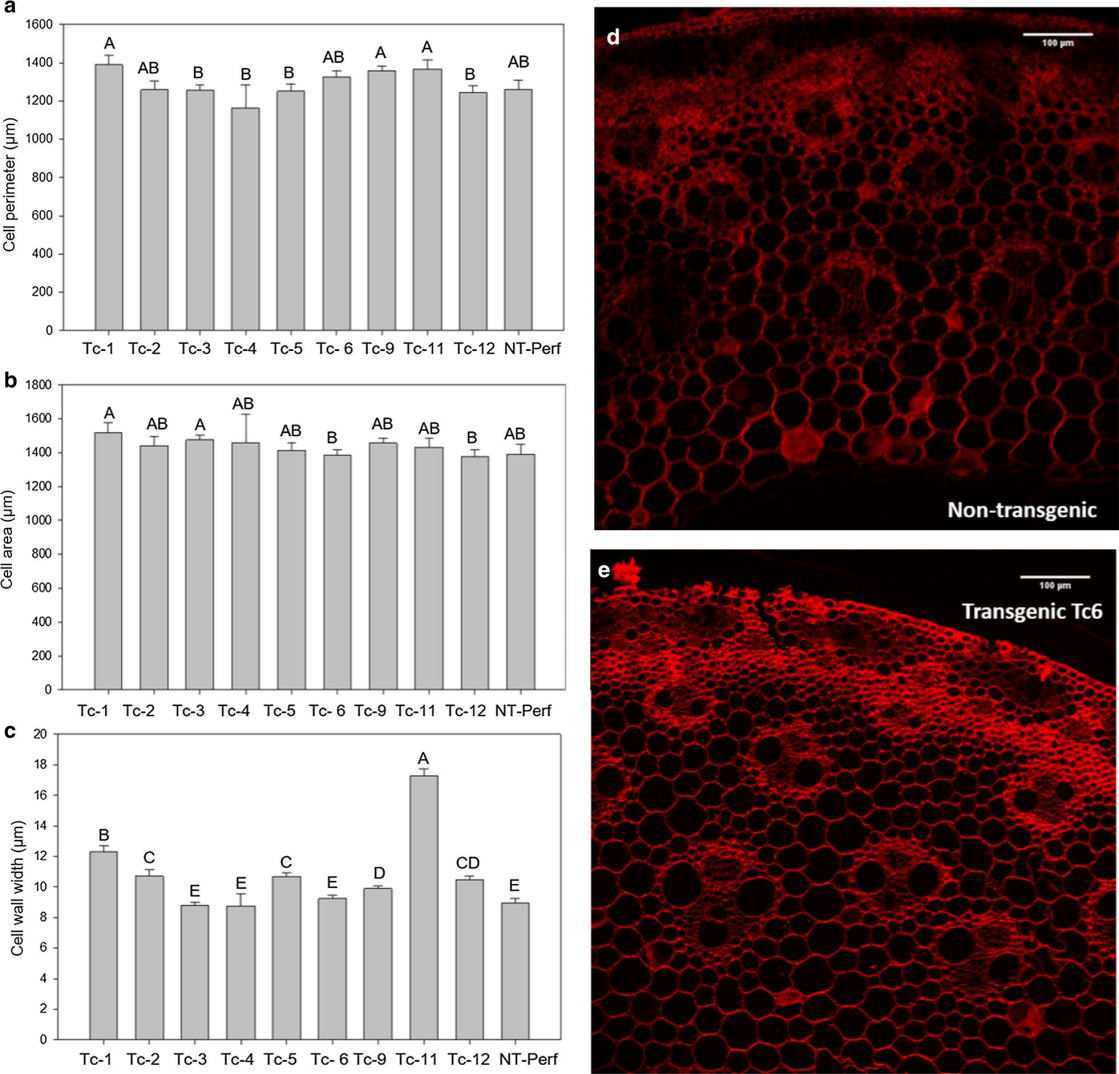
Cell wall measurements on histological analysis of stem internode sections of transgenic TcEG1 and non-transgenic (NT-Perf) plants. Measurement of cell wall perimeters (**a**), cell wall thickness (**b**), and cell wall areas (**c**). Representative images of non-transgenic (**d**) and transgenic event Tc-6 (**e**) stem internodes stained with Pontamine Fast Scarlet. Bars represent mean value of replicates ± standard error. Bars represented by different letters are significantly different as calculated by LSD (*p* ≤ 0.05). Scale bar represents 100 µm

**Fig. 8:**
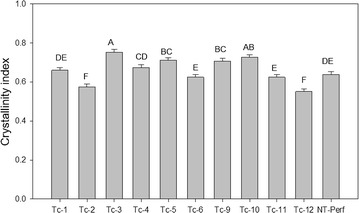
Cellulose crystallinity index measurements for transgenic TcEG1 and non-transgenic (NT-Perf) plants. Bars represent mean values of three replicates ± standard error. Bars represented by different letters are significantly different as calculated by LSD (*p* ≤ 0.05)

Histological analysis of stem internode sections revealed no differences in cell wall area or cell wall perimeter among plants (Fig. 7a, b). Transgenic events Tc-1, Tc-2, Tc-5, Tc-9, Tc-11, and Tc-12 had increased cell wall thickness by up to 93% in event Tc-11 with an average overall increase of 37% over the control (Fig. 7c). Cellulose index of crystallinity was increased by up to 18% in events Tc-3, Tc-5, Tc-9, and Tc-10, decreased by up to 10% in events Tc-2 and Tc-12, and was unchanged in events Tc-1, Tc-4, Tc-6, and Tc -11 relative to the control (Fig. 8).

### Plant morphology and growth was minimally affected by TcEG1 production

**Fig. 9:**
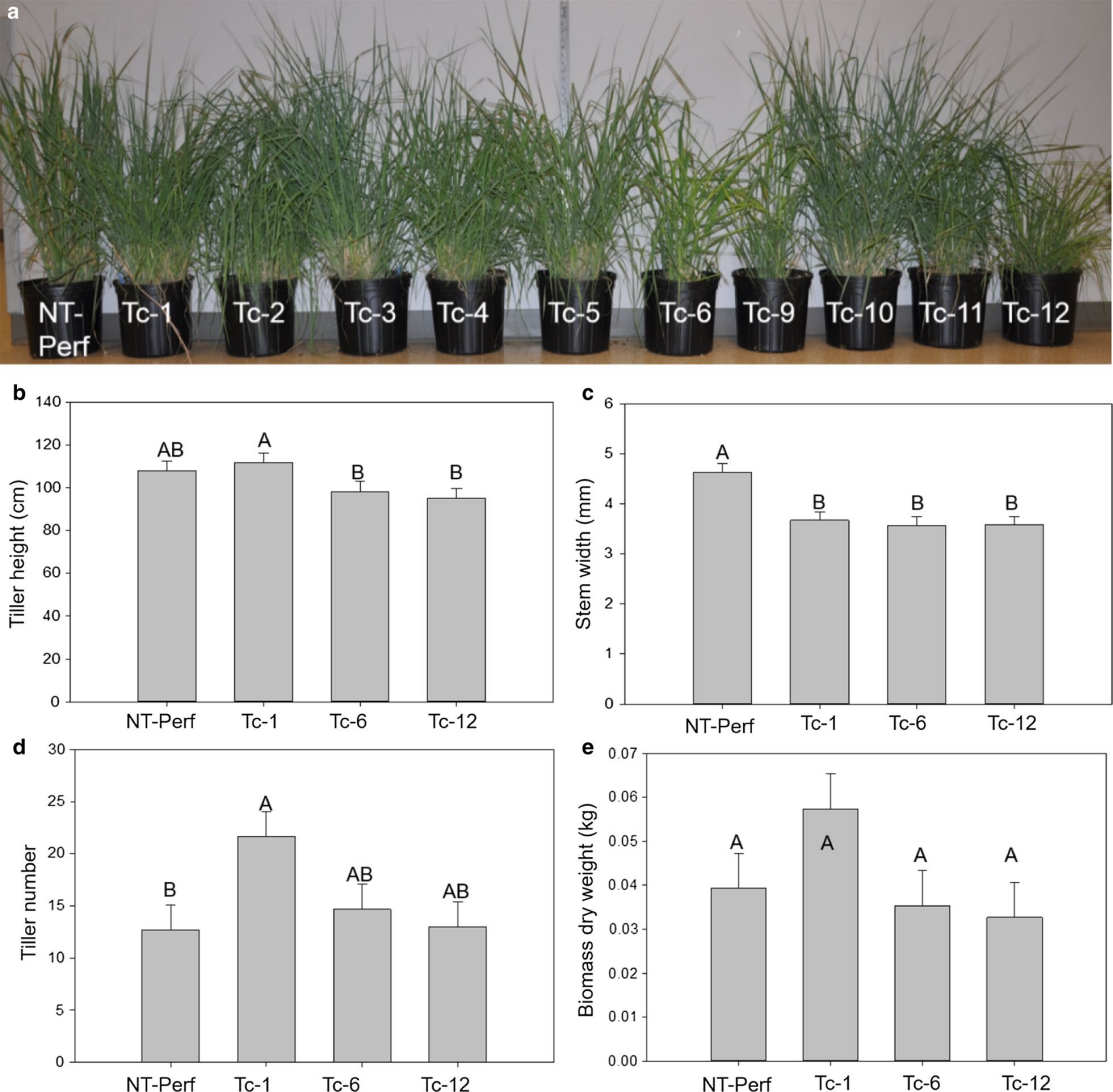
Plant morphology analysis of transgenic TcEG1 and non-transgenic switchgrass plants. **a** Representative transgenic TcEG1 and non-transgenic (NT-Perf) lines. Tiller height (**b**), stem width taken at 10 cm height above potting mixture (**c**), tiller number (**d**), and biomass dry weight (**e**) of transgenic TcEG1 and non-transgenic (NT-Perf) plants. Bars represent mean values of three replicates ± standard error. Bars represented by different letters are significantly different as calculated by LSD (*p* ≤ 0.05)

The same subset of transgenic switchgrass events from the air dry and auto-hydrolysis assay was used in a growth study. Most growth characteristics of selected transgenic events were not different from one another or from the control (Fig. 9a). There were no differences in plant height or dry biomass among lines (Fig. 9b, e). Stem diameter from all transgenic events was smaller than the control (Fig. 9c). Tiller number increased by 71% for event Tc-1 whereas Tc-6 and Tc-12 had equivalent numbers of tillers as the control (Fig. 9d).

## Discussion

An engineered self-degrading feedstock would represent a significant step toward an integrated strategy for reducing costs and increasing efficiency of biofuel production [7, 41–43]. In multiple studies, transgenic overexpression of microbial cellulase genes in plants resulted in increased release of fermentable sugars [13, 41, 44, 45]. While generally unexplored, properties of insect cellulases are comparable with those from microbes (thermotolerant and acidic pH optima) rendering them as feasible heterologously produced candidates in lignocellulosic feedstocks [13, 16, 46, 47]. However, some insect gut system cellulases have been discovered to have an alkaline pH optima. The variability of insect cellulase pH range most likely arises from digestive system environments with a variable pH range of 4.0–12.0 [48, 49]. Our study describes the first instance of a transgenic feedstock expressing an insect-derived cellulase gene.

The transgenic switchgrass-produced functional TcEG1 cellulase retained its alkaline pH 12.0 optimum and thermal activity of 50 °C (Fig. 2), which is congruent with its properties when produced in S2 insect cells and in *Saccharomyces cerevisiae* [19, 50]. However, the TcEG1 endoglucanase activity from switchgrass was much lower than from these other heterologous microbial production systems, which might be caused by suboptimal plant expression conditions. For example, TcEG1 activity in our study was lower than a sugar cane-produced synthetic endoglucanase that targeted the chloroplast, endoplasmic reticulum (ER), or the vacuole. In that latter experiment, the approximate highest endoglucanase activity observed was 23 nmol/min/mg protein on a CMC substrate [51]. When compared with other putative insect cellulases, crude digestive protein extraction from *T. castaneum* was relatively low. Perhaps TcEG1 accumulation and enzymatic activity might be improved by intracellular targeting to specific organelles or even to specific tissues as has been reported when E1 and other cellulases have been produced in plants [13].

Crude extracted TcEG1 was active from fresh and dried tissues, whereas enzyme activity from oven-dried tissue was attenuated (Figs. 2, 4), which may have been caused by decreased moisture content of tissue. Moisture content has been shown to improve sugar release and cellulosic ethanol yields by up to 25% from rehydrated switchgrass and sugarcane biomass compared to air-dried biomass [52]. However, when transgenic alfalfa leaves that produced the E1-catalytic domain were dried at 50 °C, they showed no change in enzyme activity when compared to enzyme extracted from fresh leaf material [53]. However, the dried leaf extraction reported in Ziegelhoffer et al. [53] was carried out under different conditions from the fresh material, and with coincubation with exogenous cellulase and pectinase mixture [53]. The addition of exogenous cellulase might have increased the E1 yield recovered from the dried material over that of the non-cellulase extraction method used for fresh leaf material, which would be displayed as increased enzyme activity. Overproduced heterologous cellulase from transgenic maize and rice seeds is active after drying according to several studies [54–57]. However, fresh seed was not tested to compare if drying affected the enzyme activity. Green switchgrass harvested mid-season under forage production systems that is field-dried for at least a week has biomass moisture content of ~ 25% [58, 59], which we simulated by air drying in our experiments showing a degree of feasibility of a green tissue auto-hydrolytic system in switchgrass.

In a biorefinery scenario, the feasibility of auto-hydrolysis was assessed for several TcEG1 switchgrass lines on bulk biomass. Each of the three lines we tested produced significantly increased free cellobiose, with two lines producing nearly twice the cellobiose after 1-h incubation over the control (Fig. 5a). The release of free glucose (Fig. 5b) was not different than the control, which was not unexpected since TcEG1 is an endoglucanase predominately acting on internal cellulose bonds to release cellobiose and not glucose [10–12]. The lack of continual increase in cellobiose over time is also not surprising as excess free cellobiose has been shown to be an inhibitor on endoglucanase activity [60, 61]. The addition of β-glucosidases to break down cellobiose would be needed to determine the catalytic longevity of recombinant enzyme produced in the TcEG1 events. Breeding TcEG1 lines with other lines producing additional classes of hydrolytic enzymes may be one potential strategy for engineering auto-hydrolytic feedstock.

Saccharification with pretreatment resulted in increased sugar release only in event Tc-6 (Fig. 3), which also had lower lignin content and an increased S/G ratio (Fig. 6). Saccharification was increased up to 15% in E1 transgenic maize and tobacco at the E1’s optimal pH 5.0 [62]. Although the saccharification of TcEG1 switchgrass was unchanged in most events, it is important to consider that our saccharification experiments were performed at pH 5.0 [31] in which TcEG1 is minimally active (Fig. 2b). TcEG1 switchgrass could be incorporated with use of alkaline pretreatment methods that have been shown to remove lignin without degrading soluble sugars and potentially reduce the exogenous cellulase load needed for complete hydrolysis [63]. TcEG1 switchgrass could be also used as a crossing partner with low-lignin switchgrass, such as COMT and MYB4 transgenic lines modified for decreased lignin and modified S/G ratio and increased sugar release efficiency [64–67] to further improve the saccharification efficiency by transgene stacks.

Since the production of any cellulase *in planta* might potentially have off-effects in plant cells, we analyzed transgenic stem internode cell structure via histology. TcEG1 switchgrass cell morphology did not appear to be different than the control in cell wall area or perimeter; however, cell wall thickness was increased (Fig. 7c). While not assessed in our study, it is possible that the cytoplasm volume was reduced in these cells. The majority of histological examinations of other hydrolase expressing plants has mostly been to determine proper organelle targeting of the enzymes [62–70]. In a few cases, some phenotypic alterations have been observed. For example, rice plants overexpressing a native exoglucanase gene, EXG1, had an extra lacunae which was not observed in the controls [71]. Tobacco plants with constitutive expression of TrCel5A had increased numbers of small vessels in the stems [72]. The morphology of TcEG1 switchgrass appeared to be normal.

The increased cell wall thickness (Fig. 7c) of TcEG1 switchgrass may be the result of altered cellulose structure. Similar cell wall thickening has been observed in *Arabidopsis* overexpressing an endoglucanase from aspen (*PttCel9A1*) in which there was decreased cellulose crystallinity [73]. Cellulose crystallinity is a metric that describes the crystalline structure compactness of cellulose polymer chains. High cellulose crystallinity is negatively associated with cellulose hydrolytic capacity [74]. However, TcEG1 transgenic switchgrass had a range of cellulose crystallinity with no correlation with transgene expression or enzyme production patterns (Fig. 8). The increased cell wall thickness could have been caused by an overabundance of other cell wall components that were not examined here, for example, tightly bound cell wall sugars that may be unaccounted for during saccharification. The resultant thicker cell walls of transgenics may have been a factor that led to equivalent biomass of transgenics relative to controls even through their tillers were smaller.

Transgenic TcEG1 switchgrass plants had more tillers with narrower stem thickness, but these changes resulted in no effect on biomass production (Fig. 9). While not observed here, negative pleiotropic effects have been observed in transgenic plants that produce glycosyl hydrolases including reduced height, wrinkled leaves, and sterility [45, 53, 71, 72]. Transgenic potato plants that produced E1 under the control of a constitutive promoter were deformed when grown at 35 °C and moderately high irradiance (450 µmol quanta/m^2^/s), but when the temperature was decreased to 25 °C with lower irradiance (200 µmol quanta/m^2^/s), the plants grew normally [45]. When E1 was targeted to the chloroplast, no adverse growth was observed at 35 °C and high light intensity in potato [45]. E1 is a thermophilic enzyme whose activity was likely attenuated with the decreased temperature restoring normal phenotype. Possibly TcEG1 activity is attenuated in switchgrass as the pH of plant cells is approximately neutral [75, 76], where TcEG1 activity is low, thus preventing deleterious growth effects.

Transgenic tobacco producing the endoglucanase TrCel5A from the bacterium *Trichoderma reesei*, driven by constitutive CaMV 35S promoter, was dwarfed and had active endoglucanase [71]. When TrCel5A expression was controlled by the ethanol inducible promoter, alcR, transgenic plants produced active enzyme, but with no resultant change in plant phenotype compared with controls [71]. Furthermore, when TrCel5A was targeted to the apoplast, tobacco plants were shorter and had wrinkled and necrotic leaves. ER-targeted TrCel5A plants in the same study had a curly leaf phenotype with no change to plant height [69]. These studies indicate that organelle targeting might not be sufficient to eliminate pleiotropic effects on plant growth and require coupling with non-constitutive promoter reduce effects. Nonetheless, the production of TcEG1 in switchgrass was apparently not deleterious to plant growth.

## Conclusions

This is the first study in which an active insect cellulase has been synthesized by any plant; in this case a dedicated bioenergy crop, switchgrass. TcEG1 enzyme activity was observed in all ten independent transgenic events. However, the enzyme activity was decreased in oven-dried biomass compared to air-dried biomass. There was increased cellobiose release by each transgenic switchgrass event tested using an auto-hydrolysis experiment compared to the non-transgenic control. Xylose and glucose release under acidic conditions was increased in one transgenic event, which was also accompanied by the lowest amount of lignin content among the lines studied. Cellulose crystallinity was altered, but with no correlation to saccharification or enzyme activity. Transgenic plants developed thinner, but more, tillers than the control, and had thicker cell walls. Altogether, transgenic lines did not differ from controls in dry biomass production. Improving genetic engineering strategies by plant codon optimization and organelle targeting could increase transgenic heterologous cellulase gene yield and efficacy, which has been noted in other glycosyl hydrolase plant production reports. While the dedicated bioenergy feedstock field is nascent, we see yet another potential option for feedstock auto-hydrolysis in the expression of insect cellulolytic genes in plants.

## Additional file

**Additional file 1.** Additional figures and table.

## Abbreviations

TcEG1: *Tribolium castaneum* endoglucanase 1
ZmUbi1: maize ubiquitin 1
PvUbi1: *Panicum virgatum* ubiquitin 1 promoter and intron
PvUbi2: *Panicum virgatum* ubiquitin 2 promoter and intron
LB: left border
Hph: hygromycin B phosphotransferase coding region
35S T: 35S terminator sequence
AcV5: epitope tag
NOS T: *Agrobacterium tumefaciens nos* terminator sequence
R1: *attR1* recombinase site 1
R2: *attR1* recombinase site 2
RB: right border
Kan^r^: kanamycin resistance gene
ColE1: origin of replication in *E. coli*
pVS1: origin of replication in *A. tumefacien*
OCS T: octopine synthase terminator sequence
OFP: orange fluorescent protein
pporRFP: *Porites porites* orange fluorescent protein
R1: reproductive developmental stage 1
EDTA: ethylenediaminetetraacetic acid
PMSF: phenylmethane sulfonyl fluoride
BSA: bovine serum albumin
DNSA: dinitrosalicylic acid
CMC: carboxymethyl cellulose
ANOVA: analysis of variance
LSD: least significant difference
HPLC: high performance liquid chromatography
py-MBMS: pyrolysis molecular beam mass spectrometry
FAA: formaldehyde:ethanol:acetic acid
EtOH: ethanol
FTIR: Fourier transform infrared
ATR: attenuated total reflectance
T_0_: initial transgenic generation
S/G: syringyl to guaiacyl lignin monomer ratio
E1: *Acidothermus cellulolyticus* endoglucanase 1
ER: endoplasmic reticulum
COMT: caffeic acid 3-*O*-methyltransferase EC 2.1.1.68
MYB4: R2R3-MYB transcription factor
EXG1: *Oryza sativa* exoglucanase 1
TrCel5A: *Trichoderma reesei* cellobiohydrolase 5A
PttCel9A1: *Populus tremula* L. × *tremuloids Michx* cellulase 9A1
CaMV35s: cauliflower mosaic virus 35s promoter
