# Supplementary material for: The TcEG1 beetle (Tribolium castaneum) cellulase produced in transgenic switchgrass is active at alkaline pH and auto-hydrolyzes biomass for increased cellobiose release
Source: Biotechnol Biofuels. 2017 Nov 30;10:230. doi: 10.1186/s13068-017-0918-6 (PMC5707894; doi:10.1186/s13068-017-0918-6)
Supplement: Supplementary file 1 — Additional file 1. Additional figures and table. [file 13068_2017_918_MOESM1_ESM.docx]

**The TcEG1 beetle (*Tribolium castaneum)* cellulase produced in transgenic switchgrass is active at alkaline pH and auto-hydrolyzes biomass for increased cellobiose release**

**ADDITIONAL FILE:** Additional table and figures:

Table S1: Amino acid pBLAST of TcEG-1 protein against the switchgrass proteome database. Identity (%ID) is the percent identical. Length is the amino acid alignment coverage of TcEG-1 the switchgrass protein. Mismatches represents the lack of match of two protein sequences. Gaps are the openings in the alignment represent where no matches occurred. E value represents initial score value determined by (<https://phytozome.jgi.doe.gov/pz/portal.html>). Bit score represents log transformed score based on (<https://phytozome.jgi.doe.gov/pz/portal.html>).

| **Switchgrass protein** | **%ID** | **Length** | **Mismatches** | **Gaps** | **E value** | **bit score** |
| --- | --- | --- | --- | --- | --- | --- |
| Pavir.J22089.1 | 38.05 | 452 | 252 | 10 | 3.00E-86 | 276 |
| Pavir.Fa01623.1 | 38.16 | 456 | 237 | 9 | 4.00E-86 | 277 |
| Pavir.Ea00142.1 | 37.13 | 474 | 247 | 13 | 4.00E-86 | 281 |
| Pavir.Aa00742.1 | 39.57 | 460 | 228 | 13 | 1.00E-84 | 274 |
| Pavir.Ca00745.1 | 36.23 | 461 | 251 | 8 | 3.00E-84 | 271 |
| Pavir.Eb00189.1 | 36.79 | 473 | 248 | 11 | 4.00E-84 | 275 |
| Pavir.Ga00244.1 | 38.84 | 466 | 236 | 13 | 3.00E-83 | 272 |
| Pavir.Ab02908.1 | 37.96 | 461 | 247 | 13 | 2.00E-81 | 264 |
| Pavir.Ab02962.1 | 38.36 | 464 | 228 | 12 | 3.00E-81 | 265 |
| Pavir.Aa00400.1 | 37.74 | 461 | 248 | 12 | 5.00E-81 | 263 |
| Pavir.Cb01721.1 | 36.81 | 470 | 254 | 11 | 1.00E-80 | 266 |
| Pavir.Aa03559.1 | 36.74 | 479 | 256 | 10 | 4.00E-80 | 261 |
| Pavir.J05126.1 | 37.25 | 459 | 248 | 11 | 4.00E-79 | 258 |
| Pavir.Bb02664.2 | 38.53 | 462 | 242 | 12 | 5.00E-79 | 258 |
| Pavir.Ab00278.1 | 36.4 | 489 | 254 | 12 | 5.00E-78 | 258 |
| Pavir.Bb02664.1 | 38.66 | 463 | 240 | 12 | 2.00E-77 | 257 |
| Pavir.Aa00303.1 | 36.82 | 459 | 250 | 11 | 9.00E-77 | 252 |
| Pavir.J35890.1 | 35.65 | 474 | 265 | 10 | 1.00E-75 | 249 |
| Pavir.Fa01564.1 | 36.47 | 447 | 241 | 12 | 3.00E-75 | 248 |
| Pavir.Fb01149.1 | 36.64 | 453 | 244 | 12 | 4.00E-75 | 249 |
| Pavir.Db01736.1 | 37.34 | 466 | 249 | 12 | 1.00E-74 | 247 |
| Pavir.Ca00497.1 | 35.96 | 470 | 252 | 12 | 4.00E-74 | 248 |
| Pavir.Bb02635.1 | 34.62 | 465 | 260 | 12 | 6.00E-74 | 245 |

|  |  |  |  |  |  |  |
| --- | --- | --- | --- | --- | --- | --- |
| Pavir.Db00162.1 | 36.44 | 461 | 256 | 10 | 6.00E-73 | 243 |
| Pavir.Gb00020.2 | 36.23 | 472 | 239 | 13 | 9.00E-73 | 247 |
| Pavir.Gb00020.2 | 29.47 | 190 | 105 | 5 | 1.00E-14 | 77.8 |
| Pavir.Gb00020.1 | 36.23 | 472 | 239 | 13 | 1.00E-72 | 247 |
| Pavir.Gb00020.1 | 29.47 | 190 | 105 | 5 | 1.00E-14 | 77.8 |
| Pavir.Ea01014.1 | 33.89 | 478 | 258 | 9 | 3.00E-71 | 238 |
| Pavir.Ba01232.2 | 38.6 | 399 | 209 | 8 | 2.00E-67 | 229 |
| Pavir.Ba01232.1 | 38.6 | 399 | 209 | 8 | 2.00E-67 | 229 |
| Pavir.Fb00818.1 | 33.75 | 477 | 250 | 13 | 9.00E-66 | 223 |
| Pavir.J09205.1 | 35.26 | 397 | 226 | 6 | 9.00E-65 | 219 |
| Pavir.Da01211.1 | 36.43 | 420 | 214 | 11 | 2.00E-64 | 218 |
| Pavir.Bb01897.1 | 46.77 | 263 | 138 | 2 | 6.00E-64 | 213 |
| Pavir.Fa02209.1 | 32.83 | 463 | 270 | 10 | 8.00E-64 | 218 |
| Pavir.J03155.1 | 35.85 | 463 | 243 | 13 | 9.00E-64 | 218 |
| Pavir.J36442.1 | 35.84 | 413 | 219 | 11 | 6.00E-63 | 214 |
| Pavir.J35848.1 | 32.55 | 467 | 272 | 11 | 1.00E-62 | 215 |
| Pavir.J01061.1 | 31.75 | 463 | 274 | 10 | 1.00E-61 | 212 |
| Pavir.Ia00651.1 | 34.02 | 485 | 257 | 16 | 4.00E-60 | 211 |
| Pavir.Ib04298.1 | 33.81 | 485 | 258 | 16 | 2.00E-59 | 208 |
| Pavir.Ia03435.1 | 34.77 | 463 | 232 | 16 | 1.00E-57 | 202 |
| Pavir.Bb02664.3 | 44.57 | 258 | 141 | 2 | 1.00E-55 | 193 |
| Pavir.Ga01268.2 | 33.2 | 485 | 254 | 16 | 3.00E-55 | 197 |
| Pavir.Ga01268.1 | 33.2 | 485 | 254 | 16 | 5.00E-55 | 196 |
| Pavir.Ga01270.1 | 31.79 | 497 | 249 | 19 | 9.00E-51 | 184 |
| Pavir.Gb01448.1 | 31.83 | 487 | 261 | 17 | 1.00E-48 | 177 |
| Pavir.Ia00651.2 | 32.95 | 440 | 237 | 14 | 5.00E-48 | 175 |
| Pavir.J07817.1 | 43.95 | 223 | 120 | 3 | 1.00E-41 | 157 |
| Pavir.J07817.1 | 27.67 | 159 | 93 | 5 | 5.00E-04 | 43.9 |
| Pavir.Ib04298.2 | 37.65 | 247 | 133 | 6 | 6.00E-37 | 142 |
| Pavir.Ib04298.3 | 37.65 | 247 | 133 | 6 | 8.00E-37 | 143 |
| Pavir.J06476.1 | 30.4 | 454 | 250 | 12 | 2.00E-35 | 138 |
| Pavir.J31800.1 | 38.87 | 247 | 130 | 7 | 1.00E-34 | 135 |
| Pavir.J16274.1 | 33.9 | 351 | 179 | 12 | 2.00E-33 | 132 |
| Pavir.J32907.1 | 48.7 | 115 | 59 | 0 | 1.00E-31 | 121 |
| Pavir.Ib01536.1 | 43.64 | 110 | 57 | 2 | 4.00E-22 | 96.3 |
| Pavir.Ga01737.1 | 26.69 | 281 | 161 | 11 | 3.00E-20 | 92 |
| Pavir.Ba02164.1 | 31.18 | 93 | 54 | 4 | 1.00E-05 | 47.4 |
| Pavir.Eb02381.1 | 38.78 | 49 | 23 | 2 | 0.033 | 35.8 |


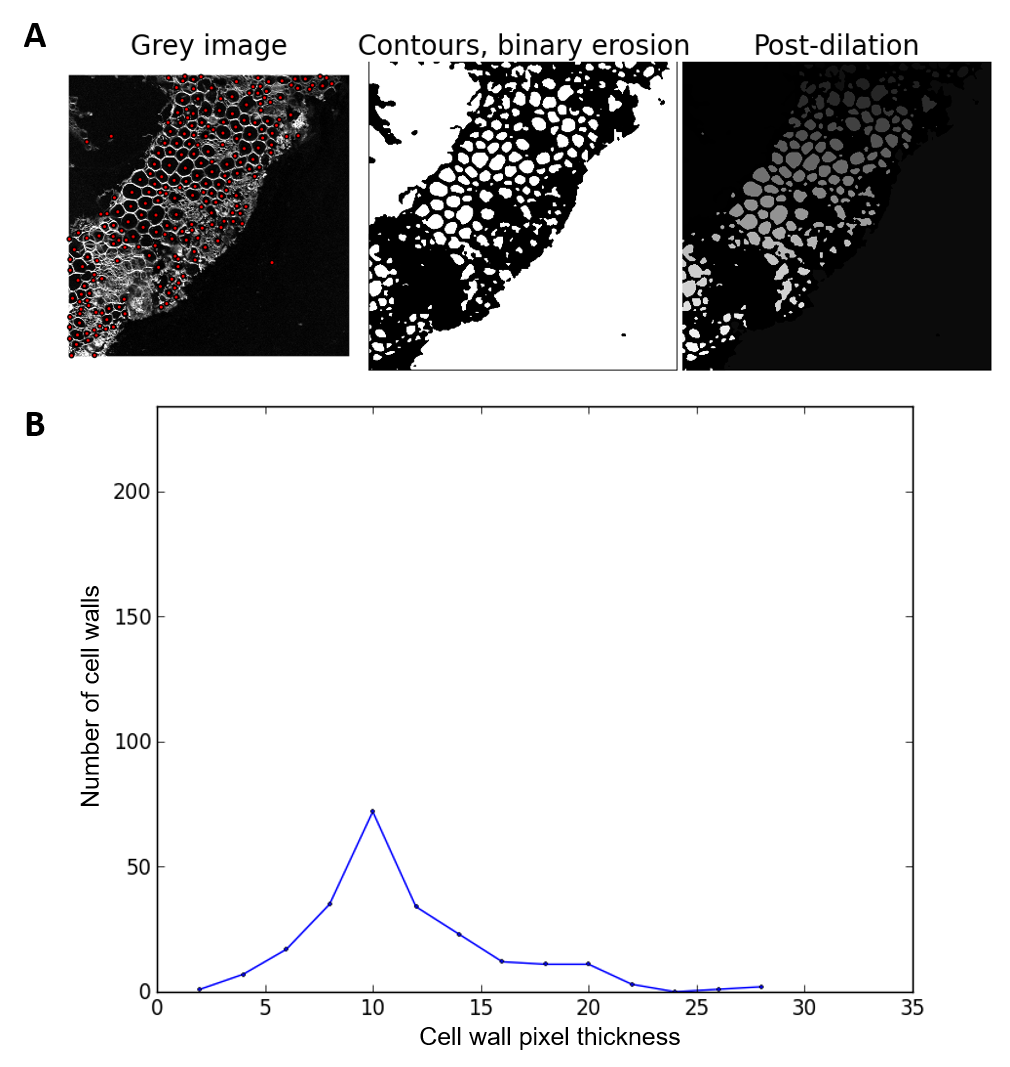


Figure S1. Stepwise demonstration of custom Python Cell Wall Thickness (pyCWT) program to identify sclerenchyma and parenchyma cells within a slide section specimen and determine output. A) Slide section images are converted to gray scale in which cells to be measured are marked with a red dot. A combined contours and binary erosion technique acts to reduce noise in the image; the final frame shows the separate regions obtained (gray scale) that were used to place red dots in the first frame. B) Example output graph of pyCWT. pyCWT calculates the number of dilations required for two cells to overlap, which is subsequently recorded. Displayed is the number of cell walls imaged against the pixel distribution. The mode was chosen as the most representative cell wall thickness for the image. The mode for each image is converted to micrometers by a separate script which extracts the micrometer size of each image from a separate text file and then performs the operation to convert pixels into micrometers for each image individually. The final output is placed into a tab-separated values file, with the following columns: image name, mode (for entire image), cell identifier, cell wall thickness, area, perimeter, and pixel-to-micron factor.


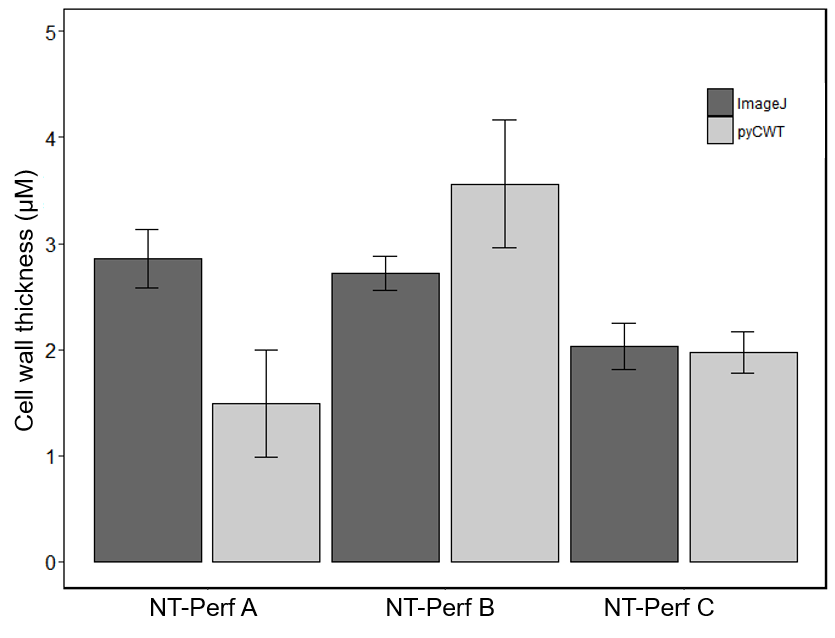


Figure S2 Comparison ImageJ manual measuring vs Python Cell Wall Thickness (pyCWT) program measuring of cell wall thickness measurement methods. Three non-transgenic switchgrass stem internodes were imaged and cell wall thickness determined by either manual measuring of cells using ImageJ or pyCWT. The Python program-generated values did not differ from the hand measured values when compared via t-test at (p < 0.05). Standard errors of the mean are shown.
